# For Motion Assistance Humans Prefer to Rely on a Robot Rather Than on an Unpredictable Human

**DOI:** 10.1109/OJEMB.2020.2987885

**Published:** 2020-04-16

**Authors:** Ekaterina Ivanova, Gerolamo Carboni, Jonathan Eden, Jörg Krüger, Etienne Burdet

**Affiliations:** ^1^ Imperial College of ScienceTechnology and Medicine 4615 London WC1E 7HT U.K.; ^2^ Technische Universität Berlin 26524 10623 Berlin Germany

**Keywords:** Physical interaction, human-human, human-robot, haptic Turing test, performance and perception

## Abstract

*Objective:* The last decades have seen a surge of robots for physical training and work assistance. How to best control these interfaces is unknown, although arguably the interaction should be similar to human movement assistance. *Methods:* We compare the behaviour and assessment of subjects tracking a moving target with assistance from (i) trajectory guidance (as typically used in robots for physical training), (ii) a human partner, and (iii) the reactive robot partner of Takagi *et al*. *Results:* Trajectory guidance was recognised as robotic, while the robot partner was felt as human-like. However, trajectory guidance was preferred to assistance from a human partner, which was recognised as less predictable. The robot partner also was felt to be more predictable and helpful than a human partner, and was preferred. *Conclusions:* While subjects like to rely on predictable interaction, such as in trajectory guidance, the control reactivity of the robot partner is essential for perceiving an interaction as human-like.

## I. Introduction

Robotic interfaces are increasingly used for work in physical contact with humans, e.g. to assist in manufacturing cars [1], to improve mobility [2], or to facilitate the training of functional tasks after a stroke [3]. Therefore, it is important to understand how users of these contact robots perceive the interaction and would like to interact with them. User perception of automated interacting partners has been analyzed in the areas of social robotics [4], humanoid robotics [5] and supported decision making [6]. The underlying applications included the handing of an object from a robot to a human [7], the remote control of autonomous robots for search and rescue [8], and robot-assisted rehabilitation, where patients and therapists were asked about their general view on the system used [9] and on new technologies’ application for the rehabilitation [10]. However, to our knowledge no previous study has examined the perception of physical interaction with a robot.

Various algorithms have been developed to control physical interaction with human movement. In current robotic interfaces, the user's movement is usually guided by the interface along some predefined reference trajectory, e.g. [11]–[13]. Such *trajectory guidance* (TG) does not consider the exchange of haptic information that takes place during human physical interaction [14]. This sensory exchange was taken into account to design the interactive *robot partner* (RP) of Takagi *et al*. [15], that can identify the user's control and adapt its own control correspondingly. Interacting with the RP was shown to yield the same motion benefits as a *human partner* (HP), in contrast to trajectory guidance [15]. However, it is not yet known how the RP affects the user's interaction behaviour and perception.

We first ask how human-like are TG and the RP. For this purpose we examined the behaviour and perception of subjects interacting with TG and the RP in comparison with a HP. In particular, we studied: (i) How these three control modalities affect the performance of subjects during motion assistance, (ii) Whether the subjects can differentiate between the interaction with a human or robotic partner, (iii) How the subjects characterise the different interaction types. Our first hypothesis (H1) is that *the RP, which incorporates the reaction to the user's movement, will be more human-like than the TG*.

Moreover, as humans perform many tasks while physically interacting with each other and are adept at it [16], we hypothesize (H2) that *the users of robotic interfaces will prefer human-like interaction*. We tested this second hypothesis by analysing (iv) Which interaction type the subjects prefer. In line with our hypothesis, we expect that the subjects prefer interacting with a HP rather than with the RP, and prefer that interaction to the TG.

**Figure 1.**
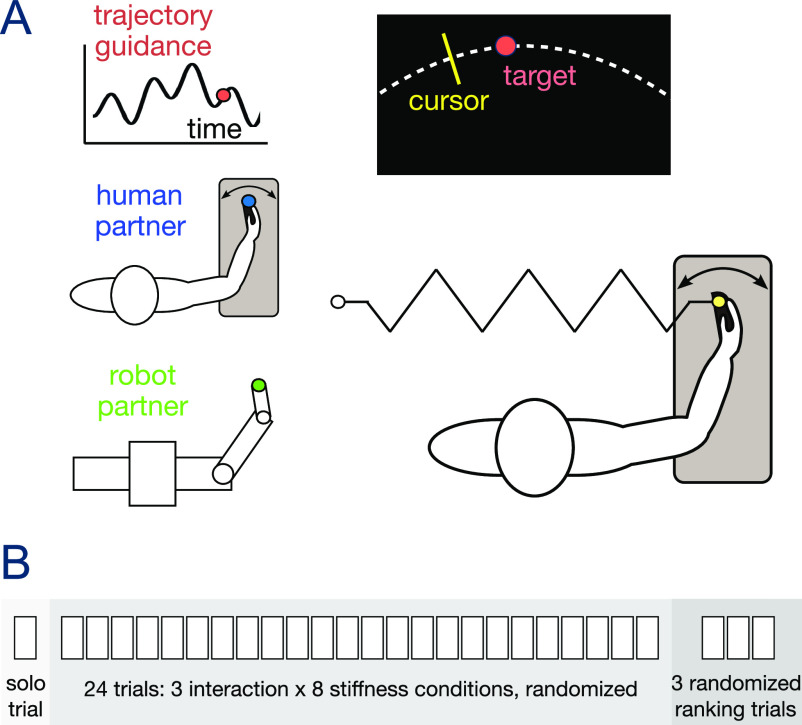
Experiment description. A: Subjects track a randomly moving target with their wrist flexion/extension movement while being connected to either trajectory guidance (TG), a human partner (HP), or a reactive robot partner (RP). The experimental protocol is shown in B.

In our experiment, healthy subjects were connected to one of three different partner types: TG, a HP or the RP, performing the same tracking task (Fig. 1A). They were asked to track a target trajectory using wrist flexion/extension movement of their dominant hand. During the motion, the Hi5 robotic interface [17] applied a torque

\begin{equation*}
\tau (t) \equiv \, \kappa \left[ q_r(t)-q(t) \right] \tag{1}
\end{equation*}

on the wrist, where $q(t)$ is their wrist flexion/extension angle and $q_r(t)$ the partner angle. In TG, $q_r(t)$ was the target trajectory, for HP it was the connected partner's wrist angle, and in the RP, $q_r(t)$ evolved according to the reactive interaction model of [15]. The connection stiffness $\kappa$ was varied between trials since this parameter has been shown to impact interaction behaviour [18]. Each subject carried out 24 trials with one of the interactions types {TG, HP, RP} at one of eight stiffness levels {0.29, 0.63, 1.03, 1.72, 2.86, 4.70, 7.73, 17.02}Nm/rad in randomised conditions. After each trial, the subjects had to answer the questions in the caption of Fig. 3 on how they felt about the interaction. The experimental protocol is summarised in Fig. 1B.

**Figure 2.**
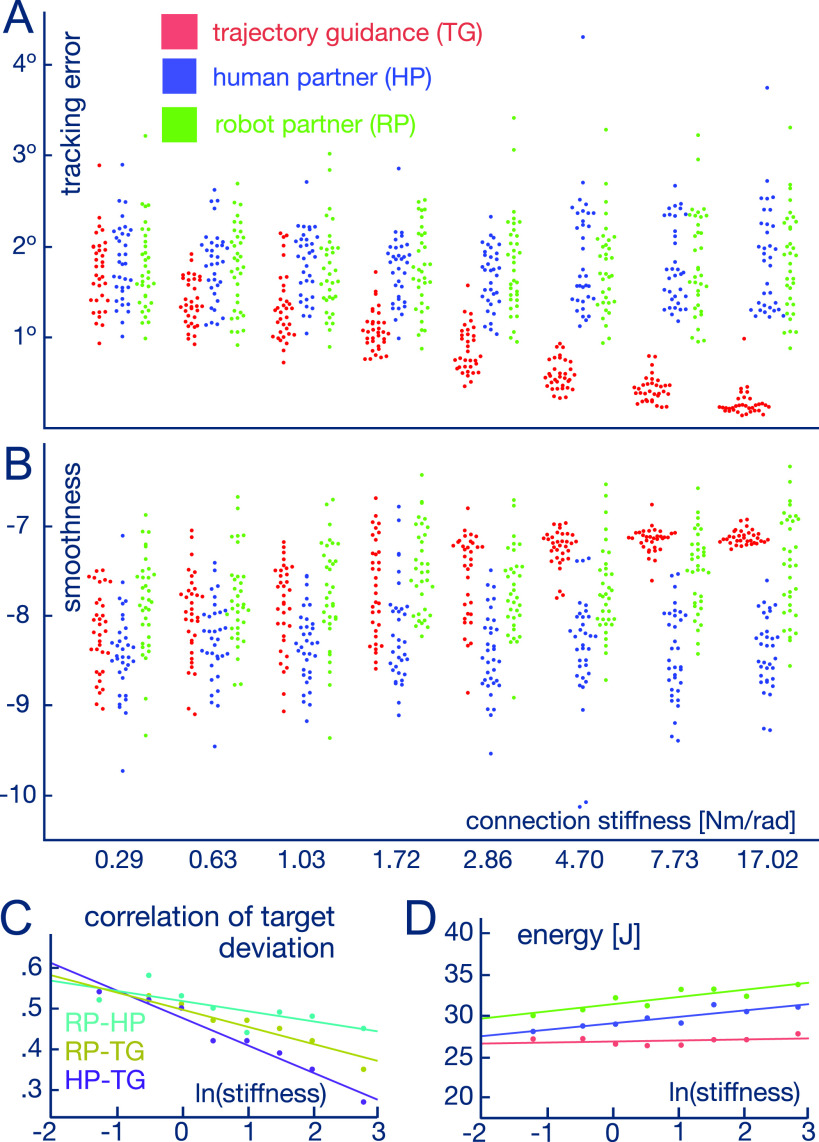
How performance depends on the coupling rigidity. A. Tracking error. B: Spectral smoothness metrics [20]. C: Correlation of signed error between the different interaction types. D: Median of energy spent for one trial, over the subjects. In all panels stiffness is in Nm/rad.

**Figure 3.**
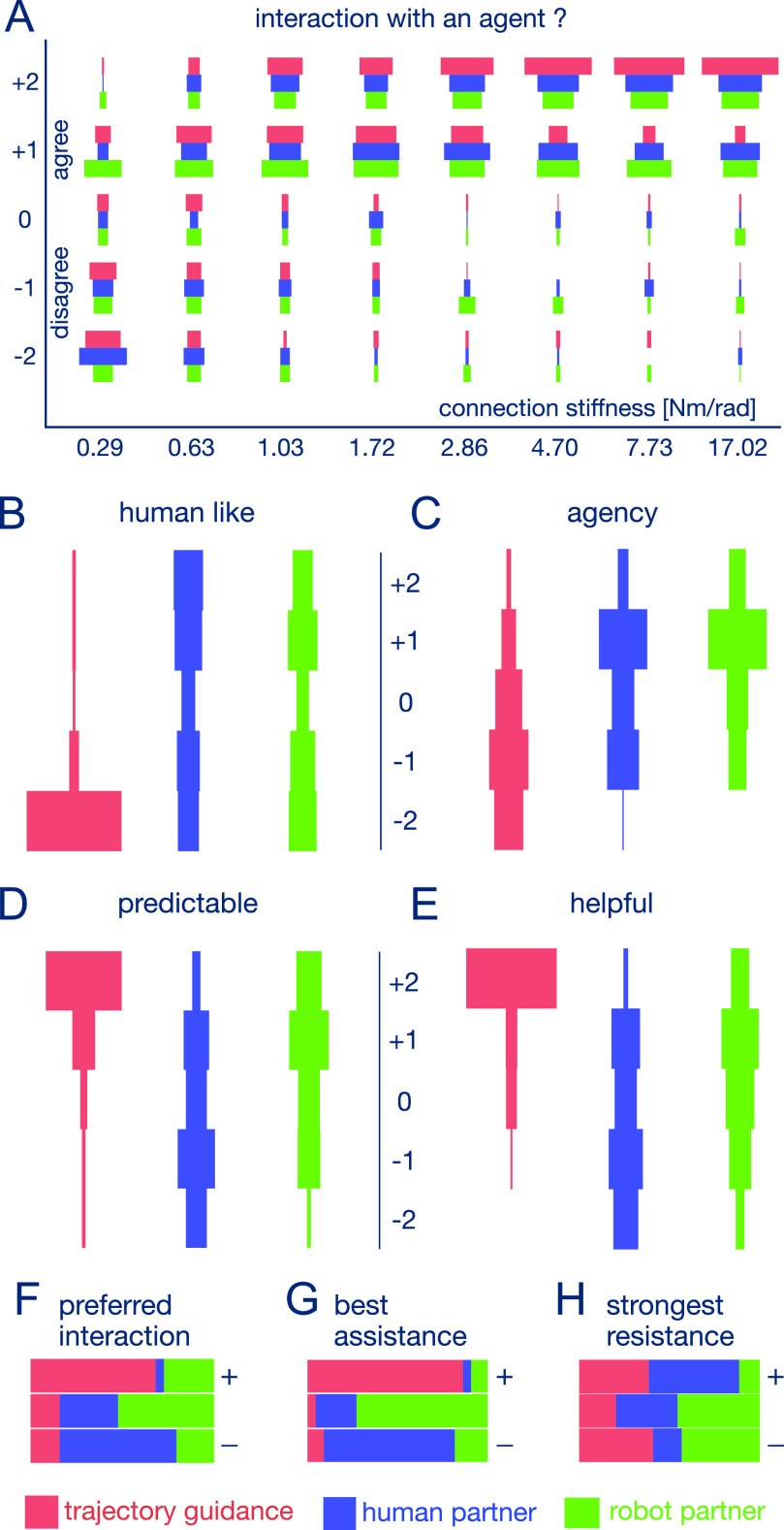
Assessment of user experience. A: How the feel of interacting with an agent depends on the connection stiffness, where −2/+2 corresponds to strongly dis/agree. B-H: Histograms of interaction assessment, where questions B-E were answered after each trial. B: Turing question “Was the interaction with a human (+2) or a robot (−2)?” C: Agency question “I felt in control of the interaction” (−2/+2: strongly dis/agree). D: “How predictable was the interaction?” (−2/+2: very un/predictable). E: “Was the interaction disturbing or helpful?” (−2/+2: very disturbing/helpful). F,G,H: Final comparison of the three interaction types according to “preference” (F), “best assistance” (G) and “strongest resistance” (H). In D-H ranking is shown from least preferred (–) to most preferred (+).

## II. Results

### A. Performance

How do the subjects perform with the different interactions? With a low stiffness connection (0.29 Nm/rad), the three interaction types result in a similar level of tracking error (Fig. 2A, *t*(33) = 2.243, *p* = 0.190 between HP and TG, *t*(33) = 0.776, *p* = 1.0 between HP and RP as well as *t*(33) = 0.582, *p* = 1.0, TG and RP, two-sample *t*-tests between all pairs with Bonferroni adjustment for multiple comparisons). When the connection becomes more rigid, as expected the TG reduces the *tracking error*

\begin{equation*}
\frac{1}{T} \! \int _0^T \!\!\! |q(t) - q^*\!(t)| \, dt\,, \tag{2}
\end{equation*}

(linear least-square fit of slope −0.374 as function of $\ln (\kappa)$, $p < $ 0.0001 relative to 0 slope), where $q(t)$ is the wrist flexion/extension angle, $q^*\!(t)$ the target trajectory and $T$ = 30s the trial duration. However with both the RP and HPs the error remains at the same level (no increase with *p* = 0.411 for RP and *p* = 0.629 for HP based on a linear least-square fit). Similarly, the variance of error decreases with increasing connection stiffness for TG (F(33, 33) = 7.572, $p < $ 0.0001 between soft and rigid connection stiffness, F-test with Bonferroni adjustment), but remains unchanged for both HPs and the RP (F(33, 33) = 0.729, *p* = 1.0 for RP and F(33, 33) = 0.558, *p* = 0.588 for HP for the difference of error between the soft and rigid connections, F-test with Bonferroni adjustment).

Furthermore, we used the *signed deviation*

\begin{equation*}
q(t)- q^*\!(t) \tag{3}
\end{equation*}

to analyse how the different interactions affect the users’ movement patterns. Fig. 2C shows that the correlation of signed deviation between TG and HP over all subjects drastically decreases with increasing stiffness. In contrast, the correlation between RP and HP only slightly decreases probably due to the oscillations of force in HPs [19]. The high correlation between the HPs and the RP indicates that the user deviates in the same way when interacting with either of these partner conditions. On the other hand, the time delay is well correlated with the error (Spearman correlation = 0.82, $p < $ 0.0001). Together these two observations suggest that the error stems mainly from the delay and systematic deviations from the target trajectory.

Motion smoothness was estimated using the spectral arc length (SPARC) metric [20]. We see in Fig. 2B how it increases with a more stiff connection for TG (linear least-square fit increase of slope 0.293 as a function of $\ln (\kappa)$, $p < $ 0.0001), but remains nearly constant for HPs (least-square fit decreasing slope −0.019 of $\ln (\kappa$), *p* = 0.404). Different to the tracking error, the smoothness for the RP slightly increases with a more rigid connection (linear least-square fit increase of slope 0.095 with $\ln (\kappa)$, *p* = 0.0001). Moreover, the motion was smoother when interacting with the RP [15] as compared with HPs (*t*(33) = 11.273, $p < $ 0.0001 for the difference considering the values at all connection stiffness levels, two sample paired *t*-test with Bonferroni adjustment).

Finally, the subjects used less *energy to complete a trial*

\begin{equation*}
\int _0^T \!\!\!\! \tau (t) \, \dot{q}(t) \, dt \tag{4}
\end{equation*}

when interacting with TG than with HPs or the RP (Fig. 2D, W = 24024, $p < $ 0.0001 for the difference between TG and HP, W = 4673, $p < $ 0.0001 for HP-RP, W = 25019, $p < $ 0.0001 for TG-RP, two-sample Wilcoxon signed-rank test with Bonferroni adjustment). *Together with the evolution of tracking error and smoothness, these results show how the subjects rely on TG to guide their movement. They also indicate that, in contrast, the HPs and the RP do not constrain the movement*.

### B. Awareness of the interaction

Can the subjects perceive the behavioural differences between interacting with TG, HPs and the RP? To address this question we had to first test if the subjects feel that “they are interacting with an agent”. The responses to this question show that with increasing connection stiffness the subjects became aware of the interaction (Fig. 3A). When the stiffness was larger than 3.00 Nm/rad the subjects clearly understood that they were interacting with an agent, for all three types of interaction (they did “agree” or “strongly agree” with this, W=2240, *p* = 0.009 for stiffness level 4.70 Nm/rad and $p < $ 0.0001 for 7.73 Nm/rad (W = 2656.5) and 17.02 Nm/rad (W = 2883.5), one-sample Wilcoxon signed-rank test with Bonferroni adjustment). Therefore, we grouped all the data points for connection stiffness larger than 3.00 Nm/rad, which we subsequently used to analyse how the subjects characterised the different interactions as described in the following.

### C. Turing test and agency

Could the subjects distinguish between interaction with a robot and a human? Fig. 3B shows that TG was clearly recognised as a robot (median = −2 with W = 1082.5, $p < $ 0.0001, one-sample Wilcoxon signed-rank test). Moreover, the score on the human likeness scale is smaller for TG than for the RP and HPs ($p < $ 0.0001 in both cases, W = 214.5 for PR, W = 218 for HP, two-sample paired Wilcoxon signed-rank test with Bonferroni adjustment). This indicates that TG was perceived as more robotic compared with the two partner conditions.

On the other hand, the distributions for HPs and the RP are not different (D = 0.081, p-value = 0.817, two sample Kolmogorov-Smirnov test). This suggests that the subjects could not distinguish between the RP and HPs. In particular, they did not appear to find the interaction with the RP more robotic than with HPs (W = 1220, *p* = 0.143, two-sample paired Wilcoxon signed-rank test with Bonferroni adjustment). While the interaction with both HPs and the RP were felt as distinctly different from TG, they were not necessarily felt as human-like. In fact the subjects’ perception of both the HPs and the RP were nearly evenly distributed between the “robot” and “human” sides, perhaps because the interaction was through a robotic interface in all cases.

The results on the question of whether the subject was in control of the interaction (agency) exhibits similar but more marked trends (Fig. 3C). With TG the subjects did not feel in control (median = −1.0), while both HPs and the RP are distinctly different ($p < $ 0.0001 in both compared to TG, W = 56 for RP, W = 198.5 for HP, two-sample paired Wilcoxon signed-rank test). However, the ratings for the RP and HPs were correlated (Goodman-Kruskal-Gamma correlation = 0.81, $p < $ 0.0001). Therefore, *the perception of the RP was similar to HPs, and distinctly different from TG*.

### D. Predictability

How did the subjects characterise the different interactions? Figs. 3D, E show that they considered the “un/predictable” and “helpful/disturbing” attributes similarly (Goodman-Kruskal-Gamma correlation = 0.779, $p < $ 0.0001), and felt that TG was more predictable than the other partners (median = 2, $p < $ 0.0001 for the comparison with HP (W = 6141) and RP (W = 3902), two-sample paired Wilcoxon signed-rank test) as well as more helpful (median = 2, $p < $ 0.0001 for the comparison with HP (W = 6676) and RP (W = 5130), two-sample paired Wilcoxon signed-rank test). The feeling for HPs and the RP were again more widespread, but this time the RP was felt to be different from HPs: It was felt as more predictable (W = 4247, $p < $ 0.0001, with two-sample paired Wilcoxon signed-rank test, with median = 1.0 for RP and −1.0 for HP) and more helpful (W = 3778.5, *p* = 0.0001, with two-sample paired Wilcoxon signed-rank test, with median = 0.0 for RP and −1.0 for HP). *Interestingly, the subjects were able to finely characterise the interaction with the RP and HPs even if they found them similar in term of agency and human-like quality*.

### E. Preference

After they had completed trials with all interaction types, the subjects compared them. For this purpose they experienced one trial with each of the three interaction types presented in a random order, and had to rank them according to specific criteria. As Fig. 3F shows, the subjects preferred TG, followed by the RP, and HPs ($p < $ 0.0001 for TG-HP, *p* = 0.006 for HP-RP, *p* = 0.084 for TG-RP, Nemenyi-test for multiple comparisons of mean rank sums).

One factor that may influence this preference patterns is the perception of assistance which was well correlated with preference (Fig. 3G, Goodman-Kruskal-Gamma correlation = 0.91, $p < $ 0.0001). TG was felt to provide more assistance than the RP (p = 0.0002, Nemenyi-test), and the RP more assistance than HPs (p = 0.015, Nemenyi-test).

Another possible reason for not liking the human interaction is that the strongest resistance was felt in this case (Fig. 3H). The RP was felt to offer the least resistance and significantly less resistance than HP (Nemenyi-test, *p* = 0.006), which may not be surprising as it was designed to take into account the user's movement during interaction [15].

Analysis of the correlation between preference and performance or perception measures further suggests that people prefer interacting with a more predictable partner and smooth movements (Goodman-Kruskal-Gamma correlation = 0.51, $p < $ 0.0001 for predictability, Spearman correlation = 0.5, $p < $ 0.0001 for smoothness).

## III. Discussion

With regards to our first hypothesis, the results show that *the interaction with TG is, and is also felt as, distinctively different from the interaction with HPs or the RP*. Interacting with HPs or the RP induce trajectories systematically biased relatively to the target trajectory followed by the TG, and require more effort. While the intrinsic variability of the user's motion is strongly reduced by TG for a rigid connection, it is not modified by interaction with another human or with the RP. Furthermore, it was shown in [15] that TG is also inducing distinctly different interaction patterns than the reactive HPs and RP.

TG, which is the interaction type typically used in current robot systems for physical training, is clearly perceived as robot-like, controlling the movement, predictable and helpful. In contrast, interaction with a human partner is characterised by large variability, unpredictability and disturbance, and is felt as not constraining the user's control. These results distinguish the reactive HPs or RP from the fixed TG interaction law. They suggest *the critical influence that reactivity in control has on the interaction performance and perception*.

Interestingly, interaction with the robot partner of [15] exhibited similar perception features than with a human partner. In this sense, *the RP is the first controller that passed a haptic Turing test on a task requiring online feedback*. Previously, the pioneering study of [21] tested a robotic control that reproduced a recorded time series of interaction forces with a human partner. That robot partner elicited a different interaction behaviour than human partners and could only be used in a repeated task such as joint reaching, not for online feedback control as in our tracking task. The study [22] identified the rhythm and smoothness necessary to induce the feeling of human-like handshaking; the robot partner resulting from such modelling was still distinguished from human partners [23], [24]. The results of [25] showed that considering the partner's input improved the human-like perception quality of a handshaking reactive control algorithm. Interestingly, as in our study, interaction with the human partner of [25] was also found to not be clearly human-like. This may indicate that additional features such as realistic human hand visual or tactile feedback may be needed to elicit human-like perception in interaction.

*The results of this study further stress the importance of in depth analysis of the perception of human-robot interaction beyond simple Turing testing.* While the RP was perceived as being as human-like as a HP and resulted in similar kinematics (as indicated by the high correlation which is in contrast to e.g. the robot partner of [21]), the subjects were able to detect fine differences in the interaction between the RP and HPs. In particular, they perceived the RP as more predictable and helpful compared to HPs. To distinguish these different characteristics, the subjects may have detected the different effort required to interact with the RP vs. HPs. Alternatively, they may use the different motion smoothness induced by interaction with the RP and HPs, as the RP model of [15] is based on smooth control while human movements exhibit fluctuations (perhaps as the result of discrete motor control [19], [26]).

A surprising finding was that subjects prefer the TG interaction, which they recognise as robotic and feel as constraining, over the more human-like and less-constraining interaction with the RP or with a human partner. They even find TG less disturbing than a human partner. This surprising result contradicts our second hypothesis as it means that *the more robotic an interaction is, the more it is liked*. Interestingly, a similar result was found in [27], where humans carrying a table with a humanoid robot preferred a caster control strategy (resulting in completely different but easily predictable movements) than a human-like strategy which they found unpredictable. What are the criteria for liking an interaction? Clearly, human-like is not a criterion, while the questionnaire's answers and performance measures suggest that smoothness, predictability and assistance may be such criteria.

## IV. Conclusion

In conclusion, the subjects were able to finely characterise different types of interaction. The TG interaction was clearly identified as robotic, as well as helpful and predictable. In contrast, the HPs and the RP, which both react to the user's behaviour, were identified as more human-like. Despite not being human-like, subjects preferred the TG, possibly because they can lean on it and decrease their own effort exploiting its predictability (Fig. 2D, effort is lower with TG than with the RP or HPs: both $p < $ 0.0001, two-sample paired Wilcoxon signed-rank test with Bonferroni adjustment). However this does not mean that TG is always a desirable interaction control strategy. In particular, it is well known that passive guidance can impede learning, e.g. in motor rehabilitation [28]. On the other hand, the RP and HPs consider the partner and do not constrain their movement, thus may be more suitable to support active learning. While the RP of [15] appears to have favourable properties for interaction control with humans, it remains to be tested how it can be applied in specific assistance and learning tasks, and which performances result from its use.

## V. Materials and Methods

The experiment was approved by the Research Ethics Committee of Imperial College London and carried out by 44 healthy adult subjects (23 female). Each subject was informed about the experiment, signed a consent form, and filled in the Edinburgh handedness inventory form [29] as well as the demographic questionnaire shown in the supplementary materials.

The subjects were then asked to track a cursor moving according to

\begin{equation*}
q^*\!(t) \equiv \, 18.51 \sin {\!\left(\frac{\pi \, t}{1.547}\right)} \sin {\!\left(\frac{\pi \, t}{2.875} \right)} \tag{5}
\end{equation*}

“as accurately as possible” using the wrist flexion/extension of their dominant hand (Fig. 1A). Prior to the experiment participants were instructed that they “may or may not interact with a human or a robot” and that “this may vary from trial to trial”. 22 pairs were selected randomly in the subjects’ population. The two subjects of each pair were placed at an individual handle of the Hi5 dual robotic interface [17] and separated by a curtain to prevent visual communication. They then conducted the experiment simultaneously with the same interaction and stiffness conditions. The soft, middle and rigid stiffness levels {0.29, 1.72, 17.02}Nm/rad of [18] were used here to allow performance comparison, and five additional intermediate levels were selected according to Fechner's law [30]. The Hi5 interface was controlled at 1000 Hz, while wrist angle data was recorded at 100 Hz.

The experiment protocol is described in Fig. 1B. In the initial familiarization trial each subject attempted the task without a haptic connection. The RP's motor noise parameter [15] was then set as the deviation observed in the subject's tracking movement during this trial, to ensure that the subject and RP have similar skill level [31]. In the main experiment, subjects carried out 24 trials, each with a different combination of the 3 interaction conditions and 8 stiffness levels, presented in a random order. Therefore each of the 44 subjects experienced all 24 possible combinations. Three additional trials with a rigid connection of 17.02 Nm/rad were conducted to compare the three interaction conditions presented in random order. The participants were then asked to rank these interactions according to their preference, assistance and resistance (Figs. 3E, F, G).

The representative questions analyzed in this paper are listed in Fig. 3's caption, while similar further questions are analyzed in [32]. Only the responses for connection stiffness larger than 3.00 Nm/rad (at which the subjects clearly recognized the interaction with an agent) are analyzed here, while results at other stiffness levels and a comprehensive correlation analysis between all computed measures are reported in [32].

A two-way repeated measures analysis of variance (ANOVA) was conducted for the continuous data metrics (trajectory error, smoothness, delay). The data distribution was first examined. A Kolmogorov-Smirnov test confirmed that in all conditions the distribution was not different from a normal distribution, except for the tracking error in the TG group with stiffness 17.02 Nm/rad. Mauchly's test for each dependent variable was conducted, and the p-values were adjusted with the Greenhouse-Geisser correction when the sphericity assumption was violated. Post-hoc *t*-test contrasts and/or F-test for equality of variances were performed with the Bonferroni correction for multiple comparisons for each variable. Least-square regression analysis was used to analyze smoothness and trajectory error.

All questionnaires used a five point ordinal Likert scale. A one-way Aligned Rank Transformation ANOVA [33] for repeated measurements was applied on the ordinal data with $\kappa >$3.00 Nm/rad, where these three rigid stiffness conditions were treated as one group. As post-hoc analysis, two-sample paired or one-sample Wilcoxon signed-rank test was conducted. The Shift-Algorithm of Streitberg and Röhmel was used to handle ties in the ordinal data. The Bonferroni correction was applied on the p-values in order to control the family-wise error rate. Kolmogorov-Smirnov test was used to compare data distributions between groups.

The Friedman-Test for unreplicated complete block design was chosen for the ranking data (preference, assistance, resistance). For post-hoc analysis, Nemenyi-Test for multiple comparisons of mean rank sums was applied.

Correlation analysis was typically conducted comparing each subject and each trial. However, the correlations for the signed deviation from the target trajectory between the different partner's conditions (Fig. 2C) were calculated using a single vector taken from each time point of trajectory for every participant. Correlations between continuous variables were performed using the Spearman method, while the Goodman-Kruskal-Gamma correlation was used for the ordinal data (attributes of interaction and ranking of interaction types).

## Appendix

Following demographic questionnaire was used in the experiment:

1) What is your gender? Male, Female, Other
2) What is your age?
3) What is the highest level of education you have completed?
4) What is your primary language? English, Other
5) Do you have some experience with haptic devices (e.g. gaming controllers, joysticks, 3D haptic controllers, robotic interface)? No, Yes: please, answer 5a and 5b
5a) What devices did you use?
5b) What did you use it for?
6) Do you play or used to play computer games? No, Yes: please, answer 6a and 6b
6a) How often do/did you play? (hours/week)
6b) What kind of games do/did you play (e.g. simulations, role-playing, sports games)?
7) Do you currently experience some pain in your hands or arms? No, Yes: please, answer 7a and 7b
7a) Where exactly? left arm/hand, right arm/hand, both hands/arms, other (specify)
7b) How strong is the pain in a scale from 0 to 10?
